# Factors associated with compliance to adjuvant hormone therapy in Black and White women with breast cancer

**DOI:** 10.1186/2193-1801-2-356

**Published:** 2013-07-30

**Authors:** Sumita S Bhatta, Ningqi Hou, Zakiya N Moton, Blase N Polite, Gini F Fleming, Olufunmilayo I Olopade, Dezheng Huo, Susan Hong

**Affiliations:** Department of Medicine, University of Chicago, 5841 S. Maryland Avenue MC 3051, Chicago, IL 60637 USA; Department of Health Studies, University of Chicago, Chicago, IL USA

**Keywords:** Medication compliance, Adjuvant hormone therapy, Breast cancer, Healthcare disparities

## Abstract

**Background:**

Studies have demonstrated lower rates of breast cancer survival for Black versus White women. Factors implicated include later stages at diagnosis, differences in tumor biology, and lower compliance rates to adjuvant hormone therapy (AHT) among Black women with hormone sensitive breast cancer. We examined factors associated with compliance to AHT among Black and White women with invasive breast cancer.

**Methods:**

Women with estrogen receptor positive (ER+), non-metastatic breast cancer were identified by the cancer registry at the University of Chicago Hospital and asked to complete a mail-in survey. Compliance was defined by self-reported adherence to AHT ≥80% at the time of the survey plus medical record verification of persistence (completion of 5 years of AHT). Logistic regression was used to determine factors associated with compliance to AHT.

**Results:**

197 (135 White and 62 Black) women were included in the analysis. 97.4% of patients reported adherence to therapy. 87.4% were found to be persistent to therapy. Overall compliance was 87.7% with no statistically significant racial difference seen (87.9% in White and 87.0% in Black, *P =* 0.87). For both Black and White women, compliance was strongly associated with both perceived importance of AHT (OR =2.1, 95% CI:1.21-3.68,*P* = 0.009) and the value placed on their doctor’s opinion about the importance of AHT (OR = 4.80, 95% CI: 2.03-11.4, *P* < 0.001)*.*

**Conclusions:**

In our cohort of Black and White women, perceived importance of AHT and the degree to which they valued their doctor’s opinion correlated with overall compliance. This suggests that Black and White women consider similar factors in their decision to take AHT.

## Introduction

An estimated 75% of breast cancers express estrogen and/or progesterone receptors. For women with these cancers, treatment with five years of adjuvant hormone therapy (AHT), either tamoxifen or an aromatase inhibitor, has been shown to decrease the risk of both breast cancer recurrence and mortality by about a third throughout the first 15 years (Davies et al. 2011). Recent data has shown that continuation of tamoxifen in women with ER + disease for 10 years produces a further reduction in recurrence and can approximately halve breast cancer mortality during the second decade after diagnosis (Davies et al. 2013; Gray et al. 2013). Despite these benefits, studies have demonstrated adherence rates to range from 41% to 72% and non-persistence (discontinuation of therapy before completion of 5 years) to range from 31% to 73%.

Previous studies have identified multiple risk factors for therapy noncompliance including extremes of age (Barron et al. 2007), concomitant antidepressant use (Demissie et al. 2001), lack of social support (Kahn et al. 2007), and side effects experienced (Lash et al. 2006; Murphy et al. 2012). Ethnic and racial variations in medication compliance have also been demonstrated. A number of studies show lower rates of compliance and an increase in mortality among ethnic minorities, particularly Black women (Patridge 2003; Kim et al. 2008; Hershman et al. 2011; Hershman et al. 2009). Studies aimed at improving compliance rates have largely focused on reducing the negative side effects of therapy, however; a number of women do not experience adverse effects and are still noncompliant. Identifying modifiable factors contributing to therapy noncompliance may have significant benefits in improving breast cancer survival.

Models from health psychology have been applied to studies of medication compliance (Kucukarslan 2012). Social cognition models such as the health belief model assumes that individuals develop beliefs that influence the interpretation of information and guide behavior (Horne & Weinman 1999). A number of studies have investigated the impact of perceived benefit of AHT on compliance rates (Barron et al. 2007; Lash et al. 2006; Grunfeld et al. 2005; Fink et al. 2004; Cluze et al. 2012; Kirk & Hudis 2008; Pellegrini et al. 2010; Thewes et al. 2005; Kimmick et al. 2009; Shelton et al. 2013). Grunfeld and colleagues found that adherers were more likely to report a belief in tamoxifen benefit whereas non-adherers were more likely to report no benefit (Grunfeld et al. 2005). In a follow-up study conducted two years after initiation of therapy, Fink and colleagues found that patients’ perceived risk versus benefit of tamoxifen was critical to sustaining adherence after the immediate threat of a breast cancer diagnosis had passed (Fink et al. 2004). Other studies demonstrate a correlation between perceived benefit and future compliance*.* In a follow-up study of elderly women conducted 5-years after initiation of therapy, Lash and colleagues found that the perceived benefit of therapy assessed at baseline corresponded to future compliance (Lash et al. 2006).

Few studies have examined the association of race with attitudes regarding adjuvant hormone therapy. Shelton and colleagues found significant differences in provider communication, patient involvement in decision making, and medical trust by race of patients (Shelton et al. 2013). However, to the best of our knowledge, no study has specifically assessed whether or not differences in perceived benefit of AHT exists between Black and White women and how this impacts overall compliance rates.

Our study sought to examine factors associated with compliance to AHT in Black and White women with ER + breast cancer at a tertiary urban medical center.

## Participants and methods

This research was approved by the University of Chicago institutional review board and patients provided written informed consent for survey participation and retrospective data analysis.

### Study sample

Women who had ER + breast cancer were identified by the cancer registry at the University of Chicago. Patients were eligible for this study if they were English speaking, under 80 years of age at initial cancer diagnosis, were no more than 10 years out from their cancer diagnosis, and had been offered hormone therapy [either tamoxifen or an aromatase inhibitor (letrozole, anastrozole, or exemestane)] as part of their post-surgery treatment. Women with stage IV breast cancer, history of cancer prior to their breast cancer diagnosis, or a known *BRCA1/2* germ-line mutation were excluded from the study.

### Survey

We designed and implemented a paper survey to assess factors associated with self-reported adherence to AHT. Patients were asked to self-report demographic variables such as race, income, and education. In addition, patients were asked questions about pre-existing chronic health conditions such as diabetes and hypertension, the number of prescription medications taken, whether or not they had insurance coverage for their medications, their total out-of-pocket cost of medications per month, and perceived risk for breast cancer recurrence. The Survey Research Lab at the University of Chicago conducted pretesting of the survey questions to assess both validity and reliability. All eligible patients (N = 381) were contacted by telephone and then mailed the 15 page self- administered survey. Regardless of survey completion, all participants were given a $10 dollar gift card.

To assess adherence, women were asked how often they missed their adjuvant hormone therapy on six categories: 1) never missed a dose; 2) missed no more than 1 dose per month; 3) missed no more than 2 doses per month; 4) missed 1–2 doses per week; 5) missed ≥3 doses per week; and 6) never took the therapy. Regardless of whether or not they chose to start AHT, participants were asked how important they thought AHT was in decreasing their risk for breast cancer recurrence (not important, a little important, moderately important, or very important). Participants were also asked how worried they were about their risk for breast cancer recurrence (very, somewhat, or not worried) and how this compared to their fears at the time of their cancer diagnosis. Other questions included how worried they were about the long-term side effects of taking AHT and concerns about their overall medication costs and the potential for AHT to interfere with their other medicines. In addition, participants were asked how heavily they weighed their doctor’s opinions in their decision to take or not take adjuvant hormone therapy (almost entirely, partly, or not at all).

### Measurements

Prior studies using both self- report and pharmacy prescription refill records have used an 80% cut-off to define adherence since <80% adherence has been associated with increased mortality (Kahn et al. 2007; Molfenter et al. 2012; Stavropoulou 2011). Thus we defined adherence in our cohort by self report of taking ≥ 80% of prescribed pills on average per month and patients were considered non-adherent if they missed greater than or equal to 3 doses per week on average (which corresponded to taking therapy less than 80% of the time per month) or if they had not initiated therapy at the time of the survey.

Persistence was defined as completion of 5 years of therapy and was verified by chart review. Chart review documentation in clinic notes from the breast surgeon, radiation oncologist, or medical oncologist were used to assess completion of 5 years of AHT. At the time of chart review, 25 women (12.6%) were in their fourth year of therapy. These women were included as having completed 5 years of AHT. We felt this was appropriate since prior studies have shown that women who have taken most of their therapy are very likely to complete therapy (Molfenter et al. 2012). We also conducted sensitivity analyses by including and then excluding these 25 women and found no difference in either persistence rates or predictors of persistence. Patients with missing data were excluded from the overall analysis.

Compliance was defined as a composite of being both adherent and persistent. Of the women who were found to be persistent, only one patient (0.7%) reported non-adherence. Therefore, our definition of compliance relies heavily on persistence.

### Statistical analysis

All statistical analyses were conducted with Stata 12.1 (StataCorp, College Station, TX, USA). Demographic characteristics and variables previously mentioned were compared for adherence and compliance rates using a Spearman’s rank correlation or Kruskal-Wallis test. Originally, adherence to hormone therapy was measured as a 6-scale ordinal outcome based on self-report. However, the categories for non-adherence were very small, and thus were combined into one category. We used logistic regression models to evaluate how perceived importance of therapy, concern about breast cancer recurrence, concern about side effects of therapy, and how heavily they valued their doctor’s opinion factored into their decision to adhere and complete therapy. Self- reported race and value placed on their doctor’s opinions were adjusted in the multivariable model to remove confounding effects (Model B) and is considered the full model. Concern about side effects was tested as an additional confounder (Model C) but was not included in the full model. To test if the associations differed by race, we generated an interaction term between race and the variable of interest, and used the Likelihood-ratio test to determine if the interaction was significant.

## Results

### Patient demographics/characteristics

Of the original 381 women who were asked to participate in the study, 226 (59.3%) completed the survey. Thirteen (6%) were excluded because they did not meet inclusion criteria and 15 (7%) were excluded from the analysis because they did not self-report race as either White or Black. Therefore survey data for 197 (87%) women (135 (68.5%) White and 62 (31.5%) Black) were available. Adherence information was available for 192 of the 197 women (5 were excluded due to missing information). Medical records were available for persistence information on 166 out of 197 women. However only 162 women had complete information on both adherence and persistence and thus we were able to assess overall compliance on 162 women.

Patient demographics including age, income, education level and co-morbid conditions are described in Table 1.

**Table 1:** **Characteristics of black and white women**

N (%)	White	Black	Total
	N = 135	N = 62	N = 197
**Age (mean; range)**^♦^	58.0; 39-72	58.1; 42-73	58.1; 39-73
**Education level**^+^			
< High school	8 (5.9%)	9 (14.5%)	17 (8.6%)
High school graduation	52 (38.5%)	33 (53.2%)	85 (43.2%)
Bachelor’s degree	31 (23.0%)	8 (12.9%)	39 (19.8%)
Graduate degree	44 (32.6%)	12 (19.4%)	56 (28.4%)
**Income**^+^			
<60 K	40 (29.6%)	38 (61.3%)	78 (39.6%)
60 K +	89 (65.9%)	16 (25.8%)	105 (53.3%)
Unknown	6 (4.4%)	8 (12.9%)	14 (7.1%)
**Comorbid Medical Conditions**^+^^			
None	52 (38.5%)	11 (17.7%)	63 (32.0%)
>1 comorbid condition	83 (61.5%)	51 (82.3%)	134 (68.0%)
**Insurance coverage**^+^			
None	0 (0%)	3 (4.8%)	3 (1.5%)
Some	33 (24.4%)	8 (12.9%)	41 (20.8%)
Most	92 (68.2%)	40 (64.5%)	132 (67.0%)
All	8 (5.9%)	10 (16.3%)	18 (9.1%)
Unknown	2 (1.5%)	1 (1.6%)	3 (1.5%)
**Out of pocket expenses**^♦^			
None	1 (0.7%)	2 (3.2%)	3 (1.5%)
<$50/month	43 (31.9%)	31 (50.0%)	74 (37.6%)
$50-100/month	43 (31.9%)	16 (25.8%)	59 (30.0%)
$100-150/month	25 (18.5%)	5 (8.1%)	30 (15.2%)
$150-200/month	5 (3.7%)	4 (6.5%)	9 (4.6%)
$ > $200/month	7 (5.2%)	1 (1.6%)	8 (4.1%)
Unknown	11 (8.2%)	3 (4.8%)	14 (7.1%)

+P-values < 0.05 by Chi-Square test.

^♦^P-values >0.05 by Chi-Square test?

^^^ Diabetes on medication, hypertension on medication, high cholesterol on medication, kidney disease, and peripheral vascular disease.

### Overall compliance/adherence

Self-report of medication adherence was high, with 187/192 (97.4%) reporting adherence to therapy (98.5% in White versus 94.9% in Black, p = 0.15). Only 5/192 (2.6%) reported either never starting AHT or missing their doses an average of ≥3 times per week. 87.3%% (145/166) were found to be persistent to therapy on chart review (88.1% in White versus 85.4% in Black, p = 0.63). Overall compliance was 87.7% (142/162) with no statistically significant racial difference seen (87.9% in White and 87.0% in Black, p = 0.87).

### Predictors of compliance/adherence

#### Race and additional demographic characteristics

In univariate analysis shown in Table 2, compliance rates were similar between White and Black women (87.9% vs. 87.0%, P = 0.63). Compared to White women, Black women, had lower education and income levels and more comorbid conditions. Black women, however, had better insurance coverage for prescription medications compared to White women. None of these variables were significantly associated with adherence or compliance (Table 2).

**Table 2:** **Compliance to adjuvant hormone therapy by patient characteristics**

	Total N	Not compliant	Compliant	OR	95% CI
Age in years					
39-50	18	1 (5.6%)	17 (94.4%)	1.0 (Ref)	
51-60	81	12 (14.8%)	69(85.2%)	0.34	0.41-2.78
61-74	61	7 (11.5%)	54 (88.5%)	0.45	0.05-3.95
Race/Ethnicity					
White	116	14 (12.1%)	102 (87.9%)	1.0 (Ref)	
Black	46	6 (13.0%)	40 (87.0%)	0.92	0.33-2.55
Insurance coverage on prescriptions					
None	2	0 (0%)	2 (100%)	1.0 (Ref)	
Some	37	5 (13.5%)	32 (86.5%)	0.46	0.05-4.28
Most	105	14 (13.3%)	91 (86.7%)	1.45	0.06-3.81
All	15	1 (6.7%)	14 (93.3%)	-	-
Family income					
<$60 K	61	5 (8.2%)	56 (91.8%)	1.0 (Ref)	
$60 K+	91	13 (14.3%)	78 (85.7%)	0.54	0.18-1.59
Education level					
< High school	15	1 (6.7%)	14 (93.3%)	1.0 (Ref)	
High School Graduate	65	7 (10.8%)	58 (89.2%)	0.59	0.07-5.21
Bachelor’s Degree	33	5(15.2%)	28(84.9%)	0.40	0.04-3.76
Graduate Degree	49	7 (14.3%)	42 (85.7%)	0.42	0.05-3.79
Comorbid Medical Conditions					
None	54	6 (11.1%)	48 (88.9%)	1.0 (Ref)	
≥1comorbid conditions	108	14 (13.0%)	94 (87.0%)	0.84	0.30-2.32
Out of pocket expenses					
None	1	0 (0%)	1 (100%)		
<$50/month	60	8 (13.3%)	52 (86.7%)	1.0 (Ref)	
$50-100/month	47	6 (12.8%)	41 (87.2%)	1.05	0.34-3.27
$100-150/month	26	2 (7.7%)	24 (92.3%)	1.84	0.36-9.36
$150-200/month	8	2(25.0%)	6 (75.0%)	0.46	0.08-2.70
>$200/month	8	0 (0%)	8 (100%)	-	-
Perceived importance of therapy					
Not important	9	4 (44.4%)	5 (55.6%)	1.0 (Ref)	
A little important	7	3 (42.9%)	4 (57.1%)	1.07	0.14-7.82
Moderately important	38	5(13.2%)	33 (86.8%)	5.28	1.05-26.6
Very important	104	7 (6.7%)	97 (93.3%)	11.1	2.42-50.8
Concern about cancer recurrence					
Not worried	51	6 (11.8%)	45 (88.2%)	1.0 (Ref)	
Somewhat worried	86	13 (15.1%)	73 (84.9%)	0.75	0.27-2.11
Very worried	24	1 (4.2%)	23 (95.8%)	0.75	0.35-27.0
Concern about side effects					
Not concerned	40	4 (10.0%)	36 (90.0%)	1.0 (Ref)	
Small concerns	50	6 (12.0%)	44 (88.0%)	0.81	0.21-3.11
Moderate concerns	42	2 (4.8%)	40 (95.2%)	2.22	0.38-12.9
Big concerns	29	8 (27.6%)	21 (72.4%)	0.29	0.08-1.09
Value placed on doctor’s opinion					
Not at all	7	6 (85.7%)	1 (14.3%)	1.0 (Ref)	
Partly	23	5 (21.7%)	18 (78.3%)	21.6	2.09-223.7
Almost entirely	131	9 (6.9%)	122 (93.1%)	81.3	8.81-750.8

#### Perceived importance of therapy

As shown in Table 3, women who perceived therapy to be “very important” were significantly more compliant than those who perceived therapy to be “not important” (adjusted OR = 9.82, 95% CI:1.75-55.1). For this 4-scale variable, per-unit increase was associated with an OR of 2.11 (95% CI: 1.21-3.68). When examining perception of hormone therapy importance by race, no difference was seen between Black and White women (*P*-interaction = 0.99*)*. Black women were as likely as White women to perceive AHT as important.

**Table 3:** **Multivariable analysis of compliance by logistic regression**

		Model A		Model B: Full model				Model C	
	OR	95% CI	*** P***	OR	95% CI	*** P***	OR	95% CI	*** P***
Perceived importance of therapy									
Not important	1.0 (Ref)			1.0 (Ref)			1.0 (Ref)		
A little important	1.00	0.13-7.48		3.40	0.17-67.8		4.12	0.17-98.9	0.16
Moderately important	5.91	1.13-30.9	0.003	5.81	0.94-35.9	0.08	5.17	0.74-36.0	
Very important	12.6	2.63-60.3		9.82	1.75-55.1		8.50	1.32-54.7	
Per-unit increase				2.11	1.21-3.68	0.009	2.18	1.24-3.84	0.007
Race									
White	1.0 (Ref)			1.0 (Ref)			1.0 (Ref)		0.54
Black	1.72	0.50-5.90	0.39	1.66	0.43-6.35	0.46	1.52	0.40-5.73	
Value doctor’s opinion									
Not at all				1.0 (Ref)			1.0 (Ref)		
Partly				13.3	0.88-201.2	0.004	17.9	1.02-315.2	0.007
Almost entirely				45.7	3.54-590.2		54.8	3.73-805.9	
Per-unit increase				4.80	2.01-11.4	<0.001	4.62	1.91-11.2	0.001
Concern about side effects									
Not concerned							1.0 (Ref)		
Small concerns							0.78	0.17-3.59	0.55
Moderate concerns							2.29	0.11-2.86	
Big concerns							0.56	0.11-2.87	

#### Compliance/adherence by value of Doctor’s opinion

Value placed on doctor’s opinion was another strong predictor of compliance. As shown in the multivariable analysis in Table 3, women who valued their doctor’s opinion heavily were significantly more compliant than those who did not (adjusted OR = 45.7, 95% CI: 3.54-590.2). Per-unit increase of this 3-scale variable was associated with an OR of 4.80 (95% CI: 2.01-11.4) for compliance.

## Discussion

We found that for both Black and White women, perceived importance of AHT in reducing their risk for developing recurrent breast cancer and the degree to which they valued their doctor’s opinion were the most important factors contributing to their overall compliance to therapy. Interestingly, education, income, concerns surrounding medication side effects, and out of pocket expenses did not appear to significantly impact reported rates of either therapy adherence or overall compliance in our cohort. Thus perception of the importance of AHT and enhanced doctor-patient communication may be important modifiable factors that can improve compliance to AHT.

In our study, patients who significantly valued their doctor’s opinions in general were more compliant to therapy. The doctor-patient relationship (Stavropoulou 2011) and doctor-patient communication (Bultman & Svarstad 2000) has been studied extensively in the adherence literature. Studies examining barriers to statin compliance have found communication patterns between patients and their clinical providers influence statin-adherence behaviors (McGinnis et al. 2007) and medical outcomes (Molfenter et al. 2012; Kaplan et al. 1989). Although a recent randomized prospective study examining the influence of educational materials on adherence to aromatase inhibitors found no difference in compliance and persistence between groups that received the educational materials versus those that did not, it is likely that educational materials alone may not be sufficient and a multidisciplinary approach is required (Yu et al. 2012). Further supporting this is a study by Shelton and colleagues which examines doctor-patient communication in patients receiving adjuvant therapy (including chemotherapy) for breast cancer. They found that enhanced provider contact increased compliance rates (Shelton et al. 2013). Thus doctor-patient communication beyond the distribution of educational materials is one possible modifiable mechanism to improve compliance.

One strength of our study is our ability to link chart review with survey responses. We were able to correlate patients’ perceived benefit of AHT and reported adherence rates with eventual completion of AHT as documented by medical records in a cohort study design, which enhances causal inference.

A limitation of our study is the use of a self-reported adherence by survey participants. We recognize that women who participate in surveys differ from those who do not (Font et al. 2012). Although our study results suggest that Black and White survey participants who perceive AHT as important are also more likely to be both adherent and compliant to therapy, it is unclear if this observation holds for nonparticipants.

Contrary to other studies examining compliance to therapy, we found no significant difference in reported adherence or overall compliance rates between Black and White women. Several explanations are possible. Although Black women in our study had lower education levels, lower income levels, and more chronic health conditions, Black women had better insurance coverage for medications compared to White women. Thus Black women who participated in our survey had greater access to medications than White women. Another possible explanation is that our study may have been underpowered. Although we had enough power to detect a greater than 15% difference in compliance rates it is possible that true compliance rates differ by less than 15%.

Given the increase in the number of oral anti-cancer medications, identifying modifiable barriers to the use of oral therapy, especially in minority women, is critical to improving survival. Prospective studies examining factors associated with non-initiation, non-adherence, and early discontinuation of adjuvant hormone therapy are underway with special attention to ethnic minorities (Neugut et al. 2012). Perceived therapy importance and doctor-patient communication on medication compliance represent modifiable variables which can improve overall cancer survival. Further prospective studies are needed to validate these findings.
